# Evolving Sensitivity Balances Boolean Networks

**DOI:** 10.1371/journal.pone.0036010

**Published:** 2012-05-07

**Authors:** Jamie X. Luo, Matthew S. Turner

**Affiliations:** 1 Centre for Complexity Science, University of Warwick, Coventry, West Midlands, United Kingdom; 2 Department of Physics, University of Warwick, Coventry, West Midlands, United Kingdom; Technical University of Madrid, Italy

## Abstract

We investigate the sensitivity of Boolean Networks (BNs) to mutations. We are interested in Boolean Networks as a model of Gene Regulatory Networks (GRNs). We adopt Ribeiro and Kauffman’s Ergodic Set and use it to study the long term dynamics of a BN. We define the sensitivity of a BN to be the mean change in its Ergodic Set structure under all possible loss of interaction mutations. Insilico experiments were used to selectively evolve BNs for sensitivity to losing interactions. We find that maximum sensitivity was often achievable and resulted in the BNs becoming topologically balanced, i.e. they evolve towards network structures in which they have a similar number of inhibitory and excitatory interactions. In terms of the dynamics, the dominant sensitivity strategy that evolved was to build BNs with Ergodic Sets dominated by a single long limit cycle which is easily destabilised by mutations. We discuss the relevance of our findings in the context of Stem Cell Differentiation and propose a relationship between pluripotent stem cells and our evolved sensitive networks.

## Introduction

The robustness of biochemical networks in noisy environments is thought to be a key property of properly functioning cells [1]–[3]. However these genetic networks are also capable of sensing and reacting to environmental [4] or internal changes [5]. We evolve insilico genetic networks to be sensitive to the loss of interactions between genes. The resulting networks are found to exhibit a similar number of excitatory and inhibitory interactions and to be comprised mainly of highly unstable limit cycles. We also note that such sensitive networks share many of the qualitative features ascribed to stem cells (a list of these features is outlined in [6] and is also described later in the article). In particular we propose that changes in the interactions between genes as a possible means for stem cells to differentiate. This hypothesis suggests that pluripotent stem cells should be more sensitive to interaction loss than differentiated cells.

Differential equation models are commonly used to understand genetic networks [7]–[10]. We utilize a Boolean approach in which both gene expression levels and time are discretised: Genes are on (1) or off (0) and time is treated as proceeding in discrete steps. We simplify the network interactions so that genes can only either up- or down- regulate other genes or have no effect on them. Despite the abstract nature of this approach it has been used to provide high level models reproducing the qualitative behaviour of the yeast cell-cycle [11], [12] and the p53-Mdm2 gene circuitry [13]. Also a variant of this model has been useful in predicting the mutant phenotypes of *Drosophilia* [14], while another variant has provided a dynamical model which explains flower development in *Arabidopsis thaliana* [15]. It is intellectually attractive in that it simplifies the state space in a manner that many experimental scientists will find intuitive and already utilise anecdotally. While noise can be incorporated into these models they are otherwise numerically deterministic, unlike nonlinear (chaotic) differential equations where the choice of, e.g. time discretisation, can affect the network behaviour at the qualitative level.

Using evolutionary algorithms on GRN models to target specific functions is becoming a common research tool in the field. This includes designing differential equation networks to form bi-stable switches and oscillators [16] and also evolving for oscillatory behaviour in continuous space, discrete time systems [17], [18].

Evolving Boolean Networks under selection has a long tradition, and has grown rapidly with the advent of modern computing. Many previous studies in this field have focused primarily on robustness in cellular function. Bornholdt and Sneppen [19], [20] consider mutational fitness by adding/removing interaction edges and evaluating fitness by the ability of the mother and daughter networks to reach the same attractor. The evolved networks are found to have shorter attractors and larger frozen components (nodes in an attractor that do not change their state) than random networks. Braunewell and Bornholdt later considered robustness to small perturbations in update times [21]. A fully stable attractor set is quickly found in few mutational steps, usually with a decreased number of attractors and an increased basin size. Boolean networks that are dynamically robust to state space perturbations was examined by Szejka and Drossel [22]. Networks were easily evolved that would return to the same attractor after single nodes were flipped from that attractor state, thus they were considered robust to noise. Further neutral mutations could then extend the basin of attraction of the main attractor. In a later study [23] they extended the fitness condition to include a response to external signals, evolving networks capable of switching between two stable attractors, under the influence of an external control node. In both of these last two studies 100% fitness was typically reached. A common theme from these studies is the ease with which solution networks were generally found, and we also find it easy in our study to evolve highly robust networks.

Robustness is a recurring theme in the Boolean Network studies cited above and yet a vast variety of definitions are mooted, concerned with many different aspects of robustness. One reason for this is the relative ease in making a definition for robustness as the retention or reproduction of a function under small perturbations. This is a valid interpretation for both robustness to noisy expression or mutational robustness. The unifying feature is the same, i.e. that attractors remain stable and unchanged. How to define sensitivity is less clear.

Sensitivity research into Boolean Networks has largely focused on the ensemble dynamics of random Boolean Networks [24]. The typical aim is to classify the dynamics of these networks into so called ordered, chaotic and critical (phase transition between ordered and chaotic) regimes based on network characteristics, including the connectivity and choice of update functions. Different sensitivity measures have been used to make these classifications including the Derrida curve [25] and the activity and sensitivity measure [26]. These measures relate to how perturbing the state in a Boolean Network affects the resulting dynamics. Thus they differ considerably from the sensitivity measure we employ later, which considers changes in the long-term dynamics of a Boolean Network resulting from permanent interaction losses.

Consider a case where under a state space perturbation the network switches from one attractor to another, we might say that the original attractor is not robust to that perturbation, but how do we quantify how sensitive or unrobust it is? This may depend on how different the new attractor is or whether other state space perturbations from the new attractor return the system to the original attractor. Such issues stem from the general lack of a definition of function for any given Boolean Network.

**Figure 1:**
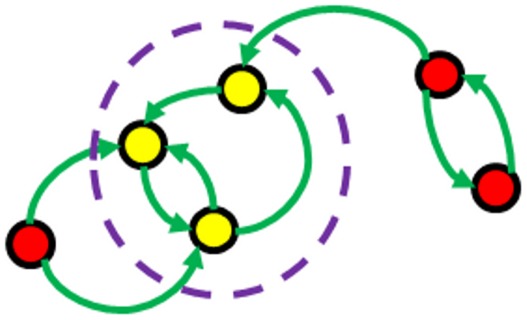
Example Ergodic Set (ES). Solid circles represent attractors of the network, whereas the arrows indicate transitions between attractors that arise when a node is flipped. Self transitions exist but are omitted from the diagram. The yellow attractors form an Ergodic Set indicated by the dashed circle. Once the system reaches an attractor in the ES it can no longer leave and so the ESs of a network represent entirely its long term behaviour. Red circles indicate the attractors which lie outside an ES.

We adopt the Ergodic Set (ES) definition proposed by Ribeiro and Kauffman [27] which incorporates both the attractors of the network and also transitions between them via state space perturbations. These perturbations take the form of single node flips from an attractor which may then result in the system moving to another attractor. An Ergodic Set is defined as a subset of attractors which is strongly connected under these transitions and has no exiting transitions (Figure 1). Thus, dynamically, if a network enters an ES it stays there under all such (weak) expression noise. Most of the Boolean Networks they examined possessed only one Ergodic Set.

Taking the ES as the basis of our definition for the global functionality of any given Boolean Network, allows us to formulate a definition for robustness and, more importantly, the sensitivity of a Boolean Network to mutations. In particular we focus on mutations resulting in the loss of an interaction between nodes. We find that evolving for sensitivity to mutations of this type leads to what we call topologically balanced networks, which have a similar number of excitatory and inhibitory interactions. The attractors of these networks are typically more unstable and this leads to sensitivity. Nearly all of the maximally sensitive networks that we find incorporate relatively long limit cycles into their ESs. These are likely to be more unstable under mutations than fixed point attractors. In addition we evolve BNs under conditions that penalise these long limit cycle cases and are still able to find networks which are highly sensitive, but non-maximal.

The ES was introduced to qualitatively describe the dynamical behaviour of a BN while accounting for the effect of noise [27]. The hypothesis that the ES of a cell defines its cell type is an extension of Kauffman’s long proposed view of the attractors of BNs corresponding to different cell types. It has also been proposed that the ES concept could prove a good qualitative model for stem cell differentiation [28]. The hypothesis here is that a single ES represents a pluripotent stem cell whereas networks with multiple ergodic sets represent differentiated cells. However very few Boolean Networks were found to possess multiple ESs and the transition from a single ES to multiple ones remained to be established. Thus in [28] was introduced the Threshold Ergodic Set (TES) concept wherein a noise parameter 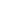 controls whether attractors are connected by the same type of transitions as defined for ESs. However a transition only exists between two attractors if there are at least a 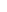 number of transitions between them. Thus changing 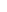 controls the number of TESs in a BN. Now multiple ESs are replaced by multiple TESs to represent differentiated cells. We propose a subtle departure from this stance which largely retains the necessary qualitative behaviour of stem cells and does not require appealing to multiple ESs or TESs. Furthermore we propose relating pluripotent stem cells to the sensitive networks we evolve.

### Model

Our Threshold Boolean Network (TBN) model represents a gene regulatory network of *N* transcriptional regulators which are represented by their gene expression patterns 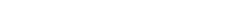 at some discrete time *t* during a biological cell process. An interaction matrix (which we also refer to as a network) 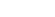 defines the regulatory interactions between genes. The entry 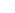 expresses the strength of interaction gene *j* has on gene *i*. We restrict ourselves to the case where 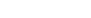. So interactions either inhibit 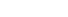 or promote 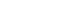 gene *i*, or are absent. Given a state 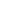 and a network *A* then the state of the system at the next time step is determined by 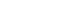 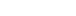 if 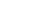 (turns on), 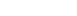 if 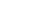 (turns off) and 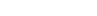 if 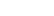 (retains the previous state). This is effectively a consensus model in the presence of multiple regulatory inputs. It should be noted that we use the common network term ‘node’ to refer to genes throughout.

For our definition of sensitivity we need to formally define an Ergodic Set (ES) of a TBN, *A*. Let *C_A_* be defined as the set of all the attractors of *A*. For a TBN these are either fixed points or finite limit cycles. Furthermore for finite systems an attractor is always reached in finite time from any initial condition. Let 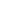 be a fixed point attractor in *C_A_*. There are *N* states in the expression space 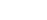 that can be reached from 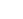 by flipping a single node. For any such flip the dynamics dictated by *A* (as detailed above) drive the system to an attractor in *C_A_* in finite time (this could be 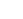 itself). All such transitions from one attractor to another (or itself) form a directed graph. Any strongly connected subset of these attractors, with no transitions that leave the subset, is defined as an ES of *A*. Figure 1 depicts an example ES. While *A* may have more than one ES, this has been found to be very rare in previous studies [27], [28] and our findings are concurrent with these. In practice we exclude instances of multiple ESs from most of our analysis, however we do acknowledge that they may be of relevance and will discuss this later.

The underlying assumption for the dynamics which define ESs is the presence of a separation in timescales between the fast deterministic TBN dynamics defined by *A* and a slow node flipping dynamic which facilitates transitions between attractors. Under this assumption, given enough time and some probability for any flip being non-zero, the ES dynamics of *A* define the long term dynamics of the network. To characterise dynamics on the ES itself we can define a Markov Chain whereby the states are attractors of the ES and the transition probabilities are defined by the flip transitions between them. We choose also to normalise these transition probabilities by the number of transitions between attractors (as multiple transitions can exist between the same two attractors) and also the length of the originating limit cycle. The second normalisation assumes an equal distribution of time between the states on any limit-cycle. Given that such a Markov Chain is ergodic by definition then we can easily calculate its stationary distribution 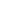 which is a probability vector over the attractor states of the ES. To complete our description of the long term dynamics on the stationary distribution, we define another stationary distribution 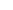 which arises when we incorporate a penalty factor in the probability transition matrix. For an attractor state 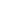 the transition resulting from flipping node *i* is normalised by the factor 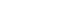 if either 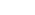 and 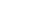 or 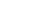 and 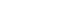 Otherwise a 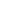 factor is used where *Z* is just a normalisation factor. The penalty factor accounts for the unlikeliness that a gene will flip on if there are other genes actively repressing it and vice versa. For a TBN with only one ES we utilise 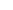 to fully characterise it’s long term dynamics.

Let there be two TBNs *A* and *B*, with ESs *E_A_* and *E_B_*, and with 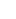 and 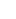 as the stationary distributions of their respective Markov Chains. We define the distance between two ESs to be 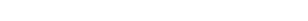 where 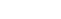 if 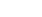 This measures how different their long term dynamics would appear in terms of the time spent on attractors inside their ESs. As we are interested in the sensitivity of *A* in relation to losing interactions, let us call 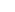 a deletion mutant of *A*. By this we mean that for some 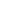 pair 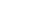 whereas 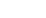 but 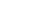 otherwise. Let 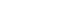 i.e. the set of all deletion mutants of *A*. The sensitivity of *A* to edge deletions is defined to be the mean of 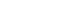 over all edge deletion mutants of *A*, i.e. 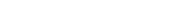 The higher the value of 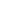 the more sensitive *A* is to edge deletions. Lower values of 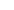 correspond to more robust networks.

The sensitivity measure 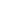 we define here bears a notable relation to the Long-run sensitivity (LRS) defined for probabilistic Boolean Networks in [29]. Our sensitivity measure corresponds to what is described in [29] as the average LRS (averaged over edge deletions in our case) in the special case of probabilistic Boolean Networks with random perturbation (defined in [29]) which only have one underlying network. In particular, by applying the small perturbation noise limit assumptions that define the ES, the average LRS value can be equivalent to our sensitivity measure 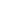 as long as two conditions are not violated. The first being trivially that *A* and its deletion mutants must each only have one ES, as otherwise 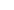 is undefined. The second requirement arises because under our 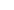 sensitivity measure, two attractors of two different ESs are considered distinct if they are different in any way, even if they share common states as can be the case with limit cycles. However the stationary distribution used in the LRS is defined over all possible states and so will assign different sensitivity contributions from the 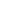 measure if an attractor in 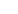 shares a common state with some non-identical attractor in some 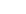


Using this new definition of sensitivity we ran insilico evolutionary experiments both to find the most sensitive networks as well as the most robust. The algorithm employed was a simple adaptive walk that started with a randomly drawn matrix *A*. At each evolutionary step a random single edge change was made to the current matrix. For instance if the edge 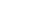 was selected for mutation, it could either be modified to 0 or −1 with equal probability for the mutant matrix 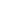 The sensitivity 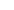 of the original was compared to the sensitivity of the altered matrix 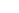 During the evolutionary search for sensitive matrices 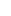 was accepted and *A* discarded if 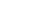 otherwise *A* was retained and a new mutation was attempted. The inequality sign is flipped for the robustness evolutions that seek to minimise 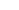 Up to 1000 mutational moves could be attempted in each run but in most cases a local fitness peak was reached well before that number. Separate evolutions were also run to find sensitive networks which did not rely solely on long limit cycles in their ESs to provide high levels of sensitivity. It should be noted that the sensitivity of networks with multiple ESs is not well defined and these rare cases were excluded from our evolutionary experiments. Pseudocode for the evolutionary experiments is included in the supporting information Text S1.

## Results and Discussion

We start by comparing the unevolved networks with their evolved counterparts for evolutions aimed at either increasing or decreasing 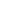 The unevolved networks form a random control ensemble. The networks evolved for robustness share similar features to previous studies. However, the sensitive networks possess two intriguing features. The first we describe as being topologically balanced, whereas the second relates to the presence of long limit cycles in their ESs, which collapse under deletion mutations. Finally we place our results for sensitive networks in the context of stem cell differentiation.

### Balance, Long Limit Cycles and Beyond

**Figure 2:**
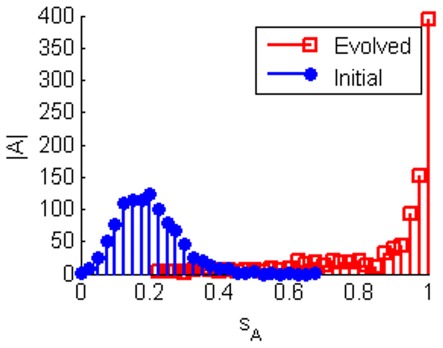
Histograms showing network sensitivity (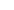). 1000 networks of 8 nodes were evolved to maximise sensitivity to edge loses. A large number achieve optimal sensitivity and almost all networks are able to improve on their initial sensitivity. 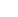 indicates the number of networks.

With regards sensitivity there are two questions we wish to address. Firstly, how sensitive can TBNs become and secondly, how do they achieve this level of sensitivity? Figure 2 shows that, even under our relatively simple evolutionary scheme, networks can often achieve optimal sensitivity through local mutations.

**Figure 3:**
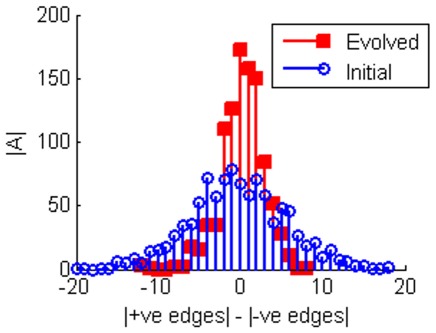
Histograms showing the edge balance for networks. The 1000 networks of 8 nodes were evolved to maximise sensitivity to edge loses. The evolved networks tend to have a more equal number of positive and negative edges than in the initial networks. The networks become more topologically balanced in order to become more sensitive to edge loses. 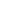 indicates the number of networks.

**Figure 4:**
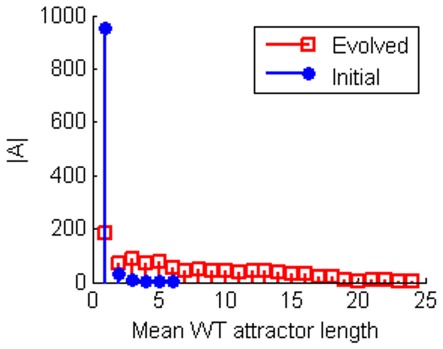
Histograms showing the average attractor length in the networks’ Ergodic Sets (ESs). The 1000 networks of 8 nodes were evolved to maximise sensitivity to edge loses. The ESs of the evolved networks tend to be dominated by very long limit cycles unlike the initial networks. These long limit cycles arise as they are more unstable under edge loses than shorter attractors. 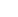 indicates the number of networks.

**Figure 5:**
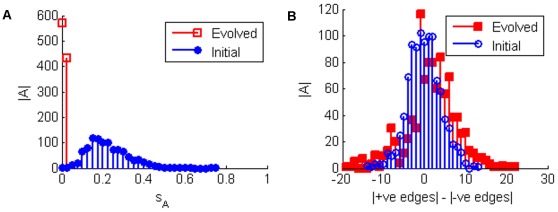
Sensitivity and Edge Balance Histograms for Robustness Evolutions. 1000 networks of 5 nodes were evolved over a maximum of 1000 generations to maximise robustness to edge losses, i.e. minimise 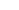 Panel A has histograms of the evolved (squares) and initial (circles) 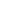 values. All evolved networks achieved at least an 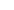 value less than 0.06. 328 networks achieved optimal robustness (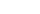). Of these 302 have an Ergodic Set (ES) with only one hoghly robust attractor, whereas the other 26 have only two attractors in their ESs. Panel B indicates that the edge balance flattens out becoming slightly more unbalanced. 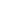 indicates the number of matrices and 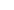 is the sensitivity as defined in the text. Looking at individual matrices reveals that typically rows become unbalanced entrenching the likelihood of the node staying on or off for the single attractor of the ES.

The primary topological trend which facilitates the increase in sensitivity is an increase in what we call topological balance. As shown in Figure 3 the number of positive and negative interactions trends strongly towards being more equal as a result of the evolution. This is consistent with the primary method by which sensitivity is achieved dynamically. In order to achieve optimal sensitivity, networks tend to evolve long limit cycle attractors, that then dominate their ESs (Figure 4). Under the TBN update rules any attractor is most unstable with respect to losing interaction edges if the number of excitatory and inhibitory inputs are balanced. Thus having an exactly balanced number of +1 and −1 edges as inputs into a node will leave an attractor most likely to be unstable with respect to losing an edge. Also, the longer the limit cycle, the more likely it is to be unstable with respect to a mutation. When evolving for robustness (i.e. smaller 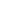), networks either achieved optimal robustness, 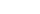 or very close to it, in the exact opposite manner to achieving sensitivity. Here the total number of edges were increased and became unbalanced (especially when looking at individual rows of an evolved matrix *A*) while the ESs were dominated by a single highly robust attractor in the vast majority of optimally robust cases (Figure 5A).

Networks with long limit cycles in their ESs that are unstable and collapse under mutations (possibly onto fixed points) is common a feature of the highly sensitive networks evolved. This mirrors the mechanism whereby the cell-cycle is arrested in response to DNA lesions [30]. If the cell-cycle is represented by a limit cycle in the ES, which in the face of deleterious mutations would collapse into fixed point attractors, then these attractors might be representative of cell cycle checkpoints. Such mappings could form the basis for using the ES and sensitivity framework presented here to discover topological features that are related to cell-cycle arrest.

**Figure 6:**
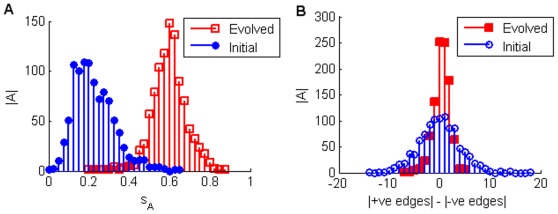
Two sets of histograms showing the sensitivity (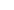) and edge balance for the networks in which long limit cycles are suppressed. The 1000 networks of 5 nodes were evolved to maximise sensitivity to edge loses, with a fitness discount factor against long limit cycles. Panel A shows that while most networks are able to increase their sensitivity (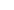) to edge loses they are unable to achieve optimal sensitivity without the longer limit cycles. Panel B shows that, as under the previous evolutionary criteria the networks become more topologically balanced in order to increase their sensitivity. 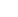 indicates the number of networks.

To investigate whether the generation of long limit cycles is the only route to greater sensitivity, further evolutionary runs were conducted with 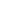 being discounted by a factor 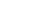
’s ES attractor length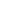. The length of an attractor is defined to be the number of states in it. *D* essentially weighs down the sensitivity score of any network whose Wild Type ES is dominated by long limit cycles. Figure 6A shows that, while no networks achieved optimal sensitivity, a substantially elevated level of sensitivity was generally achievable. In particular the 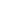 of the evolved networks is typically above 0.5. Like the previous evolved networks these ones are also topologically balanced (Figure 6B). However the ESs of these networks tend not to possess long attractors but rather fixed points instead. Note that a fixed point attractor will only stop being an attractor of *A* entirely under at most half of all possible edge losses, even if *A* is topologically balanced. So at best, losing attractors could only ever account for a 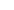 score of approximately 0.5, which is substantially below that achieved in many networks (Figure 6A). So how do these TBNs without long limit cycle achieve the additional sensitivity?

Here sensitivity arises from the mutants displacing probabilistic mass from the stationary distribution, 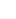 of the original or Wild Type (WT) network *A*. A mutant 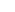 can displace this mass in the following four different ways:

1. An ES attractor of A can be made unstable (i.e. not an attractor) in 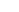

2. Mass can be placed on a new attractor included in the ES of 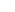 which was not in the ES of A.
3. An ES attractor of A can be retained in 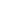 as an attractor but excluded from the ES of 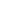

4. Mass can be redistributed across attractors which exist in both the ESs of A and 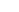

When limit cycles are not discounted against the resulting TBNs achieve sensitivity almost exclusively via methods 1 and 2. This is due to the loss of the long limit cycle from the Wild Type due to the mutation and being replaced with a new attractor in the ES of the mutant. However as noted for ESs which do not possess long limit cycles, the other methods become relevant. For the case when there are five genes, if we consider those networks with 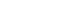 only 0.4755 of the mean 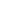 score arises from methods 1 and 2. An additional contribution of 0.0541 is gained from method 3 while a further 0.2127 is achieved through method 4. A more detailed breakdown of these statistics is provided in Table S1. These statistics show that a variety of dynamical mechanisms are employed by networks to achieve sensitivity and that this can occur in the absence of long limit cycles.

### Sensitive Networks and Stem Cells

Ergodic Sets are recognised models for stem cell differentiation [28], where the Threshold Ergodic Set 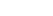 with a threshold parameter 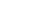 was introduced. For *A* in a 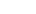 a transition from the attractor *c*_1_ to *c*_2_ only exists if at least a proportion 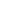 of the possible flips result in a transition from *c*_1_ to *c*_2_. For 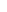 very small the ES is identical to the TES, but as 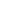 is increased the number of distinct TESs naturally increases. Varying the value of 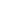 is the device used to go back and forth from a single TES to multiple TESs. By associating a single TES with the pluripotent stem cell state and multiple TESs with a differentiated stem cell, it has been argued that 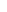 plays the role of a hidden noise parameter. In this way TESs have been used to model six distinct properties of stem cell differentiation [6]. These properties were (i) stochastic and deterministic (ii) differentiation, (iii) limited reversibility, (iv) induced pluripotency, (v) induced change of cell type and (vi) different degrees of differentiation. Here we argue instead that in most of these cases, multiple Ergodic Sets are not necessary to explain these features.

The above hypothesis relies on representing differentiated stem cells as multiple TESs and also on the varying of a parameter 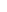 to force the appearance of multiple TESs. The need to force multiple TESs only arises though from the association of multiple TESs with a differentiated stem cell. We propose that the ES (or 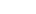) of the pluripotent cell need only be different from the ES of the differentiated cell, and that the process of differentiation can be facilitated by changes to the topology of the network. In effect we argue that stem cell networks may share dynamical properties similar to the sensitive networks we evolved earlier.

**Figure 7:**
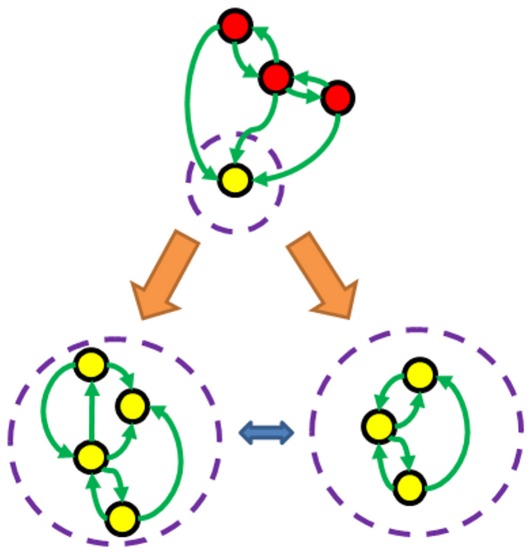
Example Ergodic Sets undergoing Differentiation via edge changes. In the three ES diagrams the circles represent attractors, with yellow ones lying inside the ES and red ones outside. Transitions are depicted by the arrows and the dashed circle marks out the ES of each network. The orange arrows indicate edge changes from the original interaction matrix. The original network was one evolved for sensitivity and as is by design its mutants have very different ES structures. The double arrow indicates that different phenotypes of differentiated cells could undergo further edge changes to switch phenotypes as is observed in experiments where cell type changes are induced.

We have already demonstrated that evolving BNs to be sensitive to edge losses is straightforward and that the ESs of the BNs are very different to those of their mutants. While we have largely referred to the loss of an interaction as a mutation it should be noted that this actually corresponds to the smallest functional change that can be enforced on a TBN type dynamical system. So if we accept the hypothesis that different ESs correspond to different cell phenotypes then a highly sensitive TBN will have the potential to differentiate, via edge changes, into a variety of cell types with distinct behaviours (Figure 7). Cell differentiation in the ES context can now be considered as a process facilitated by small topological changes to the underlying network.

**Figure 8:**
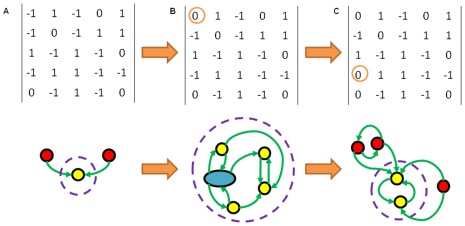
Sequence of Ergodic Sets and their Networks Modelling Stem Cell Differentiation. The interaction matrices of three Threshold Boolean Networks (TBNs) and their corresponding Ergodic Sets (ESs). In the ES diagrams the circles (and oval) represent attractors, with yellow ones lying inside the ES and red ones outside. The blue oval in B corresponds to a limit cycle of length 6. Transitions are depicted by the arrows and the dashed circle marks out the ES of each network. Network A is a five gene network evolved for sensitivity. Network B is different by one edge from A and network C is different again from B by one edge, both edge differences are encircled. Going from A-C we have a progression from a network evolved for sensitivity becoming more robust as edges are alt. Such a sequence of alterations could well correspond to the progression undergone by a cell in cases of deterministic differentiation. Thus intermediate states of differentiation like the ES of B would be candidates from which reversibility may occur going back to ES A via reversal of the network change. The induced pluripotency observed experimentally may well correspond to moving back through such a progression of network changes in a direction opposite to that indicated by the orange arrows.

Deterministic differentiation [31] can now be identified with a sequence of controlled changes to the underlying interaction network which drive the dynamics towards a desired differentiated state (Figure 8). As indicated in Figure 7 different edge changes correspond to different signals designed to achieve distinct differentiated cells. Both induced pluripotency [32], [33] and an induced change of cell type [34] are experimentally achieved by modifying the expression levels of some genes. These changes are stronger (dynamically speaking) than single edge deletions and will correspond to switching the ES of the cell either back to the plurioptent state by reversing edge changes (Figure 8) or simply shifting the system to a different differentiated state (Figure 7).

It may well be the case that pluripotency and a higher degree of sensitivity are related. Differentiated cells rarely revert to a pluripotent state, although this is possible (limited reversibility, [35], [36]). Following our hypothesis that edge changes lead to differentiation this suggests that pluripotent cells are more sensitive whereas fully differentiated cells are more robust. Indeed its possible that several edge changes are required to achieve this level of robustness from a sensitive state. This suggests that the silencing or activation of genes which lead to a sensitive pluripotent cell differentiating into a more robust differentiated cell, may correspond to unbalancing edge changes. In particular we mean changes which disturb the topological balance of a network. If differentiation corresponds to more than one edge change then limited reversibility might well be interpreted as applying to cells which are still topologically close to the pluripotent state and so can more easily switch back (for example going from network B to A in Figure 8). These cells are likely to have achieved an intermediate level of sensitivity somewhere between the highly sensitive pluripotent cells and the more robust differentiated ones. Indeed totipotent cells and other less versatile pluripotent and multipotent cells might be ranked similarly by their sensitivity. In Figure 8 the networks happen to be organised by their sensitivity (high to low from A to C). However as network A was evolved for sensitivity it is unsurprising that in most cases making edge changes away from A tends to decrease sensitivity. It is not obvious that pluripotent stem cells will lie precisely on these sensitivity peaks in the fitness landscape but we hypothesise that they may well lie on relatively higher regions than is the case when they are differentiated.

A potential testbed for our prediction that less differentiated stem cells will be more sensitive than more differentiated ones could lie with the model organism *Arabidopsis thaliana*. Insilico robustness studies performed on the *A. thaliana* root stem cell niche GRN ([37]) show that while it is reasonably robust to update function perturbations (attractors are gained or lost under about 40% of the perturbations), it is far less robust compared to other characterized GRNs responsible for cell differentiation in *A. thaliana* [38], [39]. This result coincides with our prediction on the sensitivity of stem cells. Applying our sensitivity measure to these models, perhaps under a modified form to accommodate different update rules, and exploring either their local or neutral genetic spaces may provide explanations for the difference in robustness between these networks.

We have largely ignored multiple ESs from our discussion, and from our insilico experiments, where they are deliberately excluded. This was largely because it is not mathematically obvious how our sensitivity metric functions in the presence of multiple ESs. Nonetheless, they do exist and are a piece of the puzzle which deserve further study, especially as they may well explain cases of stochastic differentiation in which identical multipotent cells stochastically generate different cell types [40], [41]. Within our picture these may correspond to edge changes that switch from a multipotent cell with a single ES to cells with multiple ESs. Evolutions were carried out in which we sought to identify for the existence of such edge changes and in almost all cases networks were found that can switch between a single and multiple ES with a single edge change. Depending on the initial state, the original cells will then switch into one of the particular ESs after such an edge change. The presence of multiple ESs in certain differentiated cells is a plausible explanation for stochastic switching between cell types.

Our conjecture that sensitive networks are related to stem cell differentiation networks suggests several avenues of further study for both theorists and experimentalists. The evolutionary methods used here are not limited to a Boolean Network formalism, however the theoretical challenge lies in constructing an appropriate fitness function. This requires an appropriate definition for the sensitivity of such a dynamical system. A modification of the description for the dynamics of stochastic differential equation systems may be a means to this end. The tendency towards topological balance that we have identified could prove a topological marker of sensitivity that may be accessible experimentally in stem cell control circuits and genetic switch systems in general.

We have proposed that many of the features of stem cell differentiation can be qualitatively replicated by edge changes resulting in changes to the underlying ES of a BN. This principle could apply more generally to other biological systems and as an example let us consider cancer. One proposal is that cancerous cells are inhabiting particular cancer attractors [42]. This proposal can be reformulated by considering cancerous ESs which might arise through mutations that alter the original cell’s ES. The challenge in applying the style of evolutionary analysis presented here to modelling the onset of cancer is in distinguishing cancer phenotypes from non-cancerous ones as expressed in ESs. Cancer does have many well documented hallmarks [43], [44] which could be utilised to define features around which evolutionary experiments could be constructed. For example cancerous cells are able to avoid apoptosis and so the process of apoptosis could be characterised by a set of attractors present in a normal cell’s ES. Evolutions could then be performed to find network’s which lose this set of attractors from the ES. The topology of such networks could then be examined, as we have done for sensitive networks, which may then reveal topological properties that characterise the greater risk for those genetic networks developing cancerous hallmarks.

## Methods

The algorithm employed to increase/decrease 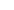 was a simple adaptive walk that started with a randomly drawn matrix *A*. At each step in the walk a random single edge change was made to the current matrix. For instance if the edge 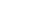 was selected for mutation, it could either be modified to 0 or -1 with equal probability for the mutant matrix 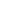 The sensitivity 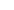 of the original was compared to the sensitivity of the altered matrix 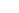 During the evolutionary search for sensitive matrices 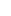 was accepted and *A* discarded if 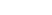 otherwise *A* was retained and a new mutation was attempted. The inequality sign is flipped for the robustness evolutions that seek to minimise 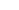 Up to 1000 mutational moves could be attempted in each run but in most cases a local fitness peak was reached well before that number. Separate evolutions were also run to find sensitive networks which did not rely solely on long limit cycles in their ESs to provide high levels of sensitivity. It should be noted that the sensitivity of networks with multiple ESs is not well defined and these rare cases were excluded from our evolutionary experiments. Pseudocode for the evolutionary experiments is included in the supporting information Text S1.

## Supporting Information

Text S1
**Pseudocode of the Sensitivity/Robustness Evolution Simulations and a Flow Chart illustrating the major steps in the Two Algorithms.** The pseudocode provides details on how the evolutionary simulations were run either to maximise or minimise 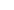 for TBNs. The flow chart illustrates both these algorithms.(DOCX)

Table S1
**Showing the Breakdown of how Probabilistic Mass is Displaced for the Evolved Networks in Sensitivity Evolutions that Penalise Long Limit Cycles.** The table shows for three system sizes (N = 5,8 and 10) a breakdown of how the probabilistic mass is displaced by the mutants of 1000 evolved networks and a select subset that meet a minimum sensitivity, 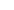. Methods 1–4 correspond to the different displacement methods mentioned in the main article. The min 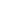 column gives the minimum sensitivity of the subset of evolved networks and the column marked No. gives the number of those networks from the originally evolved 1000 that meet the minimum sensitivity criteria.(DOCX)
