# Supplementary material for: Evolving Sensitivity Balances Boolean Networks
Source: PLoS One. 2012 May 7;7(5):e36010. doi: 10.1371/journal.pone.0036010 (PMC3346810; doi:10.1371/journal.pone.0036010)
Supplement: Text S1 — Pseudocode of the Sensitivity/Robustness Evolution Simulations and a Flow Chart illustrating the major steps in the Two Algorithms. The pseudocode provides details on how the evolutionary simulations were run either to maximise or minimise for TBNs. The flow chart illustrates both these algorithms. (DOCX) [file pone.0036010.s001.docx]

**Pseudocode for Sensitivity/Robustness Evolutionary Simulations**

The code titled **ES_v2_1_evolution_hpc.c** accurately describes how TBNs were evolved to maximize their sensitivity $s_{A}$, as described in the main text. The inequality in step 5 is reversed when evolving to minimize sensitivity (or evolve for robustness). The main idea of the code is also illustrated as a flow chart (below) for further clarity.

**ES_v2_1_evolution_hpc.c**

Inputs:

- N total number of genes (system size)
- L number of genes per cell (here L = N for single cell experiments)
- fit_param fitness parameter (sets either $\pi_{0}$ or $\pi_{1}$)
- num_gens limit on how many generations to run evolution for
- optimum_fitness sets optimum fitness achievable
- minimum_degree minimum total number of edges in a matrix (typically set to N)

Steps:

1. Generate a random initial matrix $A\in{\{-1, 0, 1\}}^{N\times N}$ with a total degree drawn from the uniform distribution between minimum_degree and $N^{2}$, set g=0, c=0
2. Call **ESdist_1mutants_del.c**(A) and record sensitivity ($s_{A}$) of the matrix
3. Mutate a single random entry of A to generate A’ (which was not previously mutated), c=c+1
4. Call **ESdist_1mutants_del.c**(A’) and record sensitivity ($s_{A'}$) of the matrix, g=g+1
5. If $s_{A'}\geq s_{A}$ then {replace A with A’ (hence $s_{A}$ with $s_{A'}$) and reset c=0}
6. If $s_{A}=$ optimum_fitness or if g=num_gens or if $c=N^{2}$ then go to step 7, otherwise go to step 3
7. Print A and $s_{A}$

**ESdist_1mutants_del.c**

Inputs:

- A matrix

Steps:

1. Find all non-zero entries in A, store in list C
2. Call **find_TES.c**(A) to find and store $ES_{A}$ (and $\pi_{0}(A)$ or $\pi_{1}(A)$)
3. For each $i\in C$do steps 3 to 6
4. Copy A into B and set entry $i$ of B to 0
5. Call **find_TES.c**(B) to find and store $ES_{B}$ (and $\pi_{0}(B)$ or $\pi_{1}(B)$)
6. Take and store ES distance $d_{ES}(\pi_{0}\left( A \right), \pi_{0}\left( B \right))$ (or $d_{ES}(\pi_{1}\left( A \right), \pi_{1}\left( B \right))$)
7. Calculate $s_{A}$ as the mean of the stored $d_{ES}$ values and return this value along with $ES_{A}$

**find_TES.c**

Inputs:

- A matrix

Steps:

1. Find the attractors of the matrix A by either exhaustive enumeration or if N is too large, find all the attractors that arise from 500 randomly drawn initial conditions.
2. From this set of attractors find all the attractors within the ESs that contain the initial set of attractors by time evolving along each single node perturbation from every attractor and recording where every transition goes. Record this weighted graph G (weighted by the number of transitions from each attractor to another).
3. Apply Tarjan’s algorithm to find all the strongly connected components of G, and check each one for whether it is an ES or not.
4. If there is found to be more than one ES terminate the entire program run and rerun from step 1 of **ES_v2_1_evolution_hpc.c**
5. Normalise the subgraph of G which comprises the unique ES_A as stipulated in the main text to compute $\pi_{0}(A)$ or $\pi_{1}(A)$ using the power method and return the appropriate vector array.

Flow Chart of Sensitivity & Robustness Evolutions
