# Supplementary material for: Evolving Sensitivity Balances Boolean Networks
Source: PLoS One. 2012 May 7;7(5):e36010. doi: 10.1371/journal.pone.0036010 (PMC3346810; doi:10.1371/journal.pone.0036010)
Supplement: Table S1 — Showing the Breakdown of how Probabilistic Mass is Displaced for the Evolved Networks in Sensitivity Evolutions that Penalise Long Limit Cycles. The table shows for three system sizes (N = 5,8 and 10) a breakdown of how the probabilistic mass is displaced by the mutants of 1000 evolved networks and a select subset that meet a minimum sensitivity, . Methods 1–4 correspond to the different displacement methods mentioned in the main article. The min column gives the minimum sensitivity of the subset of evolved networks and the column marked No. gives the number of those networks from the originally evolved 1000 that meet the minimum sensitivity criteria. (DOCX) [file pone.0036010.s002.docx]

| N | All Evolved Networks | | | | Subset of Evolved Networks with minimum s_A_ | | | | | |
| --- | --- | --- | --- | --- | --- | --- | --- | --- | --- | --- |
| Method | 1 | 2 | 3 | 4 | min s_A_ | No. | 1 | 2 | 3 | 4 |
| 5 | 0.1126 | 0.2264 | 0.0346 | 0.2255 | 0.70 | 109 | 0.1452 | 0.3303 | 0.0541 | 0.2127 |
| 8 | 0.0868 | 0.2632 | 0.0719 | 0.2471 | 0.80 | 164 | 0.0996 | 0.3853 | 0.1149 | 0.2440 |
| 10 | 0.0736 | 0.2624 | 0.0862 | 0.2547 | 0.85 | 153 | 0.0957 | 0.3903 | 0.1257 | 0.2720 |

Table S1: Displacement Breakdown of the Probabilistic Mass in Sensitivity Evolutions that Penalise Long Limit Cycles.

Notes: Methods 1-4 correspond to the different displacement methods mentioned in the main article. Min s_A_ is the minimum sensitivity of the subset of evolved networks and No. is the number of those networks from the originally evolved 1000 that meet the minimum sensitivity criteria. The table shows three system sizes, N=5,8,10.
